# *N*-(2-{[5-Bromo-2-(morpholin-4-yl)pyrimidin-4-yl]sulfan­yl}-4-meth­oxy­phen­yl)-4-methyl­benzene­sulfonamide

**DOI:** 10.1107/S1600536812033375

**Published:** 2012-07-28

**Authors:** Rajni Kant, Vivek K. Gupta, Kamini Kapoor, Mohan Kumar, L. Mallesha, M. A. Sridhar

**Affiliations:** a X-ray Crystallography Laboratory, Post-Graduate Department of Physics & Electronics, University of Jammu, Jammu Tawi 180 006, India; b Department of Studies in Physics, Manasagangotri, University of Mysore, Mysore 570 006, India; c PG Department of Studies in Chemistry, JSS College of Arts Commerce and Science, Ooty Road Mysore 570 025, India

## Abstract

In the title compound, C_22_H_23_BrN_4_O_4_S_2_, the benzene rings bridged by the sulfonamide group are tilted relative to each other by 68.9 (1)° and the dihedral angle between the sulfur-bridged pyrimidine and benzene rings is 69.7 (1)°. The mol­ecular conformation is stabilized by a weak intra­molecular π–π stacking inter­action between the pyrimidine and the 4-methylbenzene rings [centroid–centroid distance = 3.934 (2) Å]. The morpholine ring adopts a chair conformation and is disordered over two positions with an occupancy ratio of 0.853 (6):0.147 (6). In the crystal, mol­ecules are linked by N—H⋯O hydrogen bonds into chains extending along the *a* axis and further, through C—H⋯N and C—H⋯O inter­actions, into a three-dimensional supramolecular structure.

## Related literature

For the crystal structures of sulfonamides, see: Rodrigues *et al.* (2011 ▶); Akkurt *et al.* (2011 ▶). For their biological activity, see: Gao & Pederson (2005 ▶). For bond-length data, see: Allen *et al.* (1987 ▶). For ring asymmetry parameters and conformations, see: Duax & Norton (1975 ▶).
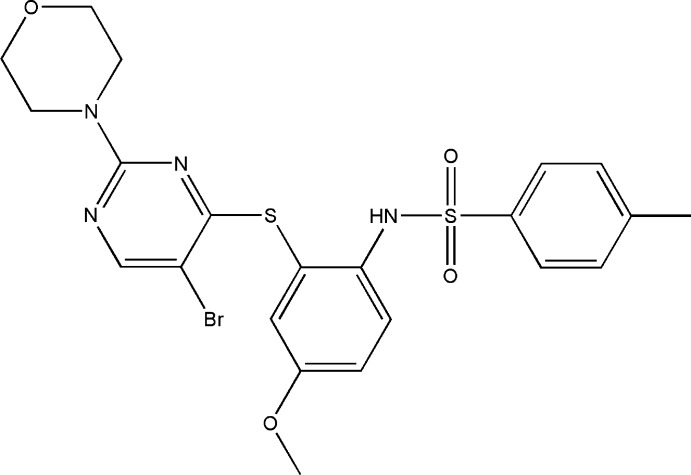


## Experimental

### 

#### Crystal data

C_22_H_23_BrN_4_O_4_S_2_
*M* *_r_* = 551.47
Monoclinic, 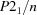

*a* = 10.0321 (3) Å
*b* = 17.4842 (6) Å
*c* = 13.9095 (4) Å
β = 91.699 (3)°
*V* = 2438.70 (13) Å^3^
*Z* = 4
Mo *K*α radiation
μ = 1.89 mm^−1^
*T* = 293 K
0.3 × 0.2 × 0.1 mm

#### Data collection

Oxford Diffraction Xcalibur Sapphire3 diffractometer
Absorption correction: multi-scan (*CrysAlis PRO*; Oxford Diffraction, 2010 ▶) *T*_min_ = 0.557, *T*_max_ = 1.000
21500 measured reflections
4783 independent reflections
3361 reflections with *I* > 2σ(*I*)
*R*_int_ = 0.040

#### Refinement

*R*[*F* ^2^ > 2σ(*F* ^2^)] = 0.041
*wR*(*F* ^2^) = 0.086
*S* = 1.01
4783 reflections
321 parameters
1 restraint
H atoms treated by a mixture of independent and constrained refinement
Δρ_max_ = 0.53 e Å^−3^
Δρ_min_ = −0.46 e Å^−3^

### 

Data collection: *CrysAlis PRO* (Oxford Diffraction, 2010 ▶); cell refinement: *CrysAlis PRO*; data reduction: *CrysAlis PRO*; program(s) used to solve structure: *SHELXS97* (Sheldrick, 2008 ▶); program(s) used to refine structure: *SHELXL97* (Sheldrick, 2008 ▶); molecular graphics: *ORTEP-3* (Farrugia, 1997 ▶); software used to prepare material for publication: *PLATON* (Spek, 2009 ▶).

## Supplementary Material

Crystal structure: contains datablock(s) I, global. DOI: 10.1107/S1600536812033375/gk2516sup1.cif

Structure factors: contains datablock(s) I. DOI: 10.1107/S1600536812033375/gk2516Isup2.hkl

Supplementary material file. DOI: 10.1107/S1600536812033375/gk2516Isup3.cml

Additional supplementary materials:  crystallographic information; 3D view; checkCIF report

## Figures and Tables

**Table 1:** Hydrogen-bond geometry (Å, °)

*D*—H⋯*A*	*D*—H	H⋯*A*	*D*⋯*A*	*D*—H⋯*A*
N8—H8⋯O26*A* ^i^	0.85 (2)	2.02 (2)	2.846 (8)	163 (2)
N8—H8⋯O26*B* ^i^	0.85 (2)	2.06 (6)	2.895 (5)	165 (3)
C21—H21⋯N20^ii^	0.93	2.53	3.394 (4)	155
C25*A*—H251⋯O2^iii^	0.97	2.59	3.377 (6)	138
C27*A*—H272⋯O2^iii^	0.97	2.52	3.341 (6)	143

Symmetry codes: (i) 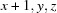; (ii) 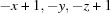; (iii) 
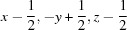
.

**Fig. 1.**
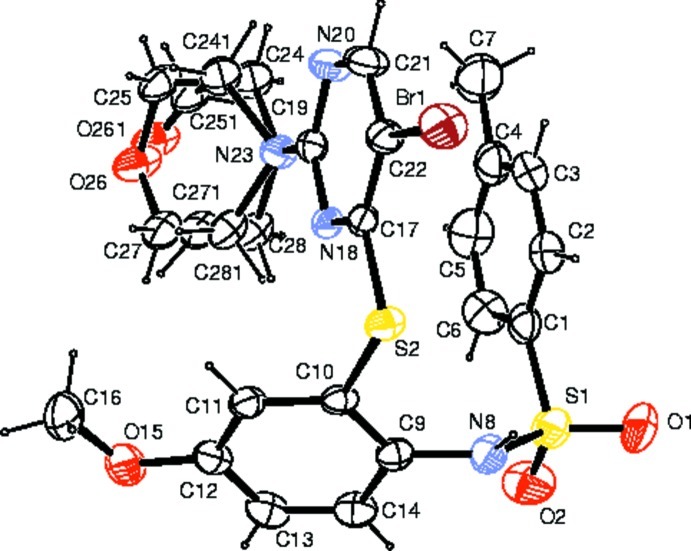
ORTEP view of the molecule with the atom-labeling scheme. The displacement ellipsoids are drawn at the 40% probability level. H atoms are shown as small spheres of arbitrary radii.

**Fig. 2.**
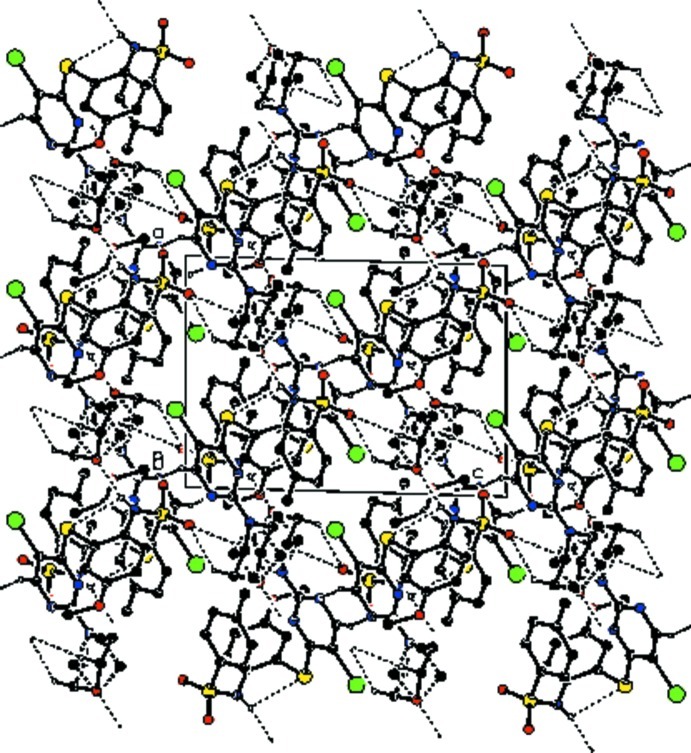
A crystal packing view of the title compound down the b axis, showing intermolecular interactions. For clarity, hydrogen atoms which are not involved in hydrogen bonding have been omitted.

## supplementary crystallographic information

### Comment

The amide and sulfonamide moieties are the constituents of many biologically significant compounds. Sulfonamide compounds are well known as antimicrobial agents (Gao & Pederson, 2005). In the present work, the crystal structure of *N*-[2-(5-bromo-2-morpholin-4-yl-pyrimidin-4-ylsulfanyl)-4-methoxy-phenyl]-4- methyl-benzenesulfonamide has been determined.

Bond lengths and angles in the title compound (Fig. 1) have normal values (Allen *et al.*, 1987) and are comparable with the similar crystal structures (Rodrigues *et al.*, 2011; Akkurt *et al.*, 2011). The morpholine ring is disordered over two positions with the occupancy ratio of 0.853 (6):0.147 (6) and adopts chair conformation (asymmetry parameters:ΔC_s_(C24A—C27A) = 1.30; ΔC_2_(C24A—C25A) = 1.59; ΔC_s_(C25B—C28B) = 2.37; ΔC_2_(C25B—O26B) = 3.65 (Duax & Norton, 1975). The two benzene rings are tilted relative to each other by 68.9 (1)°. The dihedral angle formed by the pyrimidine ring with two benzene rings (C9—C14/C1—C6) are 69.7 (1) and 2.2 (1)°, respectively. In the crystal, molecules are connected *via* N—H···O, C—H···N and C—H···O hydrogen bonds.

### Experimental

The reaction of N-[2-(5-bromo-2-chloro-pyrimidin-4-ylsulfanyl)-4-methoxy-phenyl]- 4-methyl-benzenesulfonamide (5.01 g, 0.01 mol) with morpholine (0.88g, 0.01) were carried out in the presence of triethylamine and the reaction mixture was allowed to stir at room temperature for 6-7 h in dry dichloromethane. The progress of the reaction was monitored by TLC. Upon completion, the solvent was removed under reduced pressure and the residue was extracted with ethyl acetate. The compound was purified by successive recrystallization from methanol (yield 84%, m.p. 485-487 K).

### Refinement

The N-bound H atom was located in a difference Fourier map and constrained to lie 0.86 (1) Å from the parent atom. All other H atoms were positioned geometrically and were treated as riding on their parent C atoms, with C—H distances of 0.93–0.97 Å with *U*_iso_(H) = 1.2*U*_eq_(C) or 1.5*U*_eq_(methyl C).

### Crystal data

**Table:**

C_22_H_23_BrN_4_O_4_S_2_	*F*(000) = 1128
*M**_r_* = 551.47	*D*_x_ = 1.502 Mg m^−^^3^
Monoclinic, *P*2_1_/*n*	Mo *K*α radiation, λ = 0.71073 Å
Hall symbol: -P 2yn	Cell parameters from 7219 reflections
*a* = 10.0321 (3) Å	θ = 3.5–29.0°
*b* = 17.4842 (6) Å	µ = 1.89 mm^−^^1^
*c* = 13.9095 (4) Å	*T* = 293 K
β = 91.699 (3)°	Block, brown
*V* = 2438.70 (13) Å^3^	0.3 × 0.2 × 0.1 mm
*Z* = 4	

### Data collection

**Table:**

Oxford Diffraction Xcalibur Sapphire3 diffractometer	4783 independent reflections
Radiation source: fine-focus sealed tube	3361 reflections with *I* > 2σ(*I*)
Graphite monochromator	*R*_int_ = 0.040
Detector resolution: 16.1049 pixels mm^-1^	θ_max_ = 26.0°, θ_min_ = 3.7°
ω scans	*h* = −12→12
Absorption correction: multi-scan (*CrysAlis PRO*; Oxford Diffraction, 2010)	*k* = −21→21
*T*_min_ = 0.557, *T*_max_ = 1.000	*l* = −17→17
21500 measured reflections	

### Refinement

**Table:**

Refinement on *F*^2^	Secondary atom site location: difference Fourier map
Least-squares matrix: full	Hydrogen site location: inferred from neighbouring sites
*R*[*F*^2^ > 2σ(*F*^2^)] = 0.041	H atoms treated by a mixture of independent and constrained refinement
*wR*(*F*^2^) = 0.086	*w* = 1/[σ^2^(*F*_o_^2^) + (0.0237*P*)^2^ + 2.0098*P*] where *P* = (*F*_o_^2^ + 2*F*_c_^2^)/3
*S* = 1.01	(Δ/σ)_max_ = 0.001
4783 reflections	Δρ_max_ = 0.53 e Å^−^^3^
321 parameters	Δρ_min_ = −0.46 e Å^−^^3^
1 restraint	Extinction correction: *SHELXL97* (Sheldrick, 2008), Fc^*^=kFc[1+0.001xFc^2^λ^3^/sin(2θ)]^-1/4^
Primary atom site location: structure-invariant direct methods	Extinction coefficient: 0.0044 (3)

### Special details

**Table:**

Experimental. *CrysAlis PRO*, Oxford Diffraction Ltd., Version 1.171.34.40 (release 27–08-2010 CrysAlis171. NET) (compiled Aug 27 2010,11:50:40) Empirical absorption correction using spherical harmonics, implemented in SCALE3 ABSPACK scaling algorithm.
Geometry. All esds (except the esd in the dihedral angle between two l.s. planes) are estimated using the full covariance matrix. The cell esds are taken into account individually in the estimation of esds in distances, angles and torsion angles; correlations between esds in cell parameters are only used when they are defined by crystal symmetry. An approximate (isotropic) treatment of cell esds is used for estimating esds involving l.s. planes.
Refinement. Refinement of *F*^2^ against ALL reflections. The weighted *R*-factor *wR* and goodness of fit *S* are based on *F*^2^, conventional *R*-factors *R* are based on *F*, with *F* set to zero for negative *F*^2^. The threshold expression of *F*^2^ > σ(*F*^2^) is used only for calculating *R*-factors(gt) *etc*. and is not relevant to the choice of reflections for refinement. *R*-factors based on *F*^2^ are statistically about twice as large as those based on *F*, and *R*- factors based on ALL data will be even larger.

### Fractional atomic coordinates and isotropic or equivalent isotropic displacement parameters (Å^2^)

**Table:**

	*x*	*y*	*z*	*U*_iso_*/*U*_eq_	Occ. (<1)
Br1	0.83360 (4)	0.11861 (2)	0.46770 (3)	0.06867 (15)	
S1	0.87974 (10)	0.20496 (6)	0.92605 (6)	0.0704 (3)	
S2	0.80399 (7)	0.25279 (5)	0.62864 (5)	0.0479 (2)	
O1	1.0028 (3)	0.16380 (17)	0.9297 (2)	0.0948 (9)	
O2	0.8356 (3)	0.24415 (16)	1.00940 (17)	0.0942 (9)	
C1	0.7515 (3)	0.14086 (19)	0.8915 (2)	0.0580 (9)	
C2	0.7717 (4)	0.0890 (2)	0.8185 (3)	0.0658 (9)	
H2	0.8513	0.0896	0.7858	0.079*	
C3	0.6747 (4)	0.0365 (2)	0.7942 (3)	0.0705 (10)	
H3	0.6889	0.0025	0.7441	0.085*	
C4	0.5572 (4)	0.0332 (2)	0.8422 (3)	0.0750 (11)	
C5	0.5381 (4)	0.0859 (3)	0.9135 (3)	0.0899 (13)	
H5	0.4585	0.0852	0.9462	0.108*	
C6	0.6332 (4)	0.1399 (2)	0.9384 (3)	0.0805 (11)	
H6	0.6173	0.1754	0.9865	0.097*	
C7	0.4547 (5)	−0.0272 (3)	0.8165 (4)	0.1122 (16)	
H71	0.3785	−0.0209	0.8559	0.168*	
H72	0.4926	−0.0770	0.8274	0.168*	
H73	0.4279	−0.0222	0.7500	0.168*	
N8	0.8941 (3)	0.26764 (16)	0.84004 (19)	0.0557 (7)	
C9	0.7866 (3)	0.31561 (17)	0.8096 (2)	0.0451 (7)	
C10	0.7358 (3)	0.31454 (16)	0.71564 (19)	0.0415 (7)	
C11	0.6332 (3)	0.36306 (17)	0.6863 (2)	0.0465 (7)	
H11	0.6023	0.3627	0.6226	0.056*	
C12	0.5771 (3)	0.41192 (18)	0.7516 (2)	0.0510 (8)	
C13	0.6270 (3)	0.41381 (19)	0.8452 (2)	0.0575 (9)	
H13	0.5896	0.4468	0.8894	0.069*	
C14	0.7313 (3)	0.36729 (19)	0.8733 (2)	0.0552 (8)	
H14	0.7657	0.3704	0.9361	0.066*	
O15	0.4760 (2)	0.46199 (13)	0.72933 (18)	0.0700 (7)	
C16	0.4322 (4)	0.4661 (2)	0.6311 (3)	0.0917 (13)	
H161	0.5074	0.4750	0.5915	0.138*	
H162	0.3695	0.5072	0.6229	0.138*	
H163	0.3902	0.4187	0.6127	0.138*	
C17	0.6708 (3)	0.18839 (16)	0.61096 (18)	0.0383 (6)	
N18	0.5635 (2)	0.19533 (13)	0.66305 (15)	0.0399 (5)	
C19	0.4636 (3)	0.14544 (17)	0.6460 (2)	0.0435 (7)	
N20	0.4648 (3)	0.08807 (15)	0.5818 (2)	0.0587 (7)	
C21	0.5749 (3)	0.08278 (19)	0.5312 (2)	0.0606 (9)	
H21	0.5801	0.0439	0.4858	0.073*	
C22	0.6803 (3)	0.13110 (17)	0.5425 (2)	0.0471 (7)	
N23	0.3546 (2)	0.15296 (15)	0.70003 (19)	0.0545 (7)	
C24A	0.2340 (5)	0.1087 (3)	0.6840 (4)	0.0640 (14)	0.853 (6)
H241	0.2103	0.0832	0.7430	0.077*	0.853 (6)
H242	0.2484	0.0699	0.6355	0.077*	0.853 (6)
C25A	0.1230 (4)	0.1615 (3)	0.6511 (4)	0.0714 (15)	0.853 (6)
H251	0.1428	0.1825	0.5886	0.086*	0.853 (6)
H252	0.0403	0.1329	0.6447	0.086*	0.853 (6)
O26A	0.1073 (7)	0.2244 (3)	0.7210 (6)	0.093 (2)	0.853 (6)
C27A	0.2260 (4)	0.2649 (3)	0.7336 (4)	0.0690 (14)	0.853 (6)
H271	0.2130	0.3061	0.7791	0.083*	0.853 (6)
H272	0.2497	0.2876	0.6728	0.083*	0.853 (6)
C28A	0.3385 (5)	0.2149 (3)	0.7696 (3)	0.0578 (14)	0.853 (6)
H281	0.4202	0.2444	0.7759	0.069*	0.853 (6)
H282	0.3184	0.1940	0.8321	0.069*	0.853 (6)
C24B	0.217 (4)	0.128 (2)	0.650 (3)	0.0640 (14)	0.147 (6)
H243	0.2028	0.1556	0.5906	0.077*	0.147 (6)
H244	0.2195	0.0735	0.6355	0.077*	0.147 (6)
C25B	0.117 (3)	0.143 (2)	0.711 (3)	0.0714 (15)	0.147 (6)
H253	0.0360	0.1206	0.6835	0.086*	0.147 (6)
H254	0.1368	0.1154	0.7708	0.086*	0.147 (6)
O26B	0.097 (5)	0.195 (3)	0.728 (4)	0.093 (2)	0.147 (6)
C27B	0.215 (2)	0.2360 (18)	0.795 (2)	0.0690 (14)	0.147 (6)
H273	0.1920	0.2885	0.8100	0.083*	0.147 (6)
H274	0.2302	0.2082	0.8549	0.083*	0.147 (6)
C28B	0.333 (4)	0.233 (2)	0.735 (2)	0.0578 (14)	0.147 (6)
H283	0.4109	0.2495	0.7721	0.069*	0.147 (6)
H284	0.3206	0.2667	0.6805	0.069*	0.147 (6)
H8	0.945 (2)	0.2490 (16)	0.7983 (17)	0.056 (10)*	

### Atomic displacement parameters (Å^2^)

**Table:**

	*U*^11^	*U*^22^	*U*^33^	*U*^12^	*U*^13^	*U*^23^
Br1	0.0627 (2)	0.0822 (3)	0.0620 (2)	0.00876 (19)	0.01777 (17)	−0.02139 (19)
S1	0.0824 (7)	0.0747 (7)	0.0529 (5)	0.0012 (5)	−0.0187 (5)	0.0044 (5)
S2	0.0329 (4)	0.0586 (5)	0.0525 (4)	−0.0022 (4)	0.0058 (3)	−0.0169 (4)
O1	0.0833 (19)	0.094 (2)	0.104 (2)	0.0119 (17)	−0.0426 (16)	0.0176 (17)
O2	0.146 (3)	0.095 (2)	0.0411 (13)	−0.0125 (19)	−0.0076 (14)	−0.0056 (13)
C1	0.065 (2)	0.057 (2)	0.0509 (19)	0.0106 (18)	−0.0055 (16)	0.0103 (16)
C2	0.056 (2)	0.064 (2)	0.077 (2)	0.0132 (19)	−0.0008 (18)	−0.001 (2)
C3	0.071 (2)	0.054 (2)	0.086 (3)	0.014 (2)	−0.008 (2)	−0.0039 (19)
C4	0.070 (3)	0.069 (3)	0.086 (3)	0.002 (2)	−0.007 (2)	0.018 (2)
C5	0.071 (3)	0.107 (4)	0.093 (3)	−0.004 (3)	0.018 (2)	0.015 (3)
C6	0.091 (3)	0.086 (3)	0.066 (2)	0.003 (3)	0.014 (2)	−0.001 (2)
C7	0.092 (3)	0.094 (4)	0.150 (5)	−0.019 (3)	−0.017 (3)	0.018 (3)
N8	0.0513 (16)	0.0634 (19)	0.0523 (16)	0.0050 (14)	−0.0011 (13)	−0.0056 (14)
C9	0.0397 (16)	0.0506 (19)	0.0454 (17)	−0.0066 (14)	0.0055 (13)	−0.0053 (14)
C10	0.0342 (15)	0.0445 (18)	0.0461 (16)	−0.0066 (13)	0.0063 (12)	−0.0112 (13)
C11	0.0386 (16)	0.0492 (19)	0.0515 (17)	−0.0026 (14)	−0.0006 (13)	−0.0106 (14)
C12	0.0408 (17)	0.0457 (19)	0.067 (2)	−0.0020 (15)	0.0113 (15)	−0.0096 (16)
C13	0.060 (2)	0.054 (2)	0.060 (2)	−0.0027 (18)	0.0227 (17)	−0.0143 (17)
C14	0.068 (2)	0.058 (2)	0.0398 (16)	−0.0088 (18)	0.0082 (15)	−0.0064 (15)
O15	0.0602 (14)	0.0639 (16)	0.0861 (18)	0.0172 (13)	0.0062 (13)	−0.0164 (13)
C16	0.078 (3)	0.088 (3)	0.108 (3)	0.036 (2)	−0.021 (2)	−0.021 (3)
C17	0.0350 (15)	0.0425 (17)	0.0371 (15)	0.0052 (13)	−0.0036 (12)	−0.0042 (13)
N18	0.0323 (12)	0.0437 (14)	0.0435 (13)	0.0020 (11)	−0.0002 (10)	−0.0055 (11)
C19	0.0349 (15)	0.0416 (17)	0.0540 (17)	0.0031 (14)	−0.0006 (13)	−0.0026 (14)
N20	0.0505 (16)	0.0478 (16)	0.0780 (19)	−0.0068 (13)	0.0068 (14)	−0.0197 (14)
C21	0.063 (2)	0.050 (2)	0.069 (2)	−0.0033 (18)	0.0038 (17)	−0.0260 (17)
C22	0.0452 (17)	0.0495 (19)	0.0466 (17)	0.0032 (15)	0.0032 (13)	−0.0120 (14)
N23	0.0362 (13)	0.0539 (17)	0.0735 (18)	−0.0021 (13)	0.0045 (12)	−0.0110 (14)
C24A	0.047 (2)	0.064 (4)	0.081 (4)	−0.014 (2)	0.002 (3)	0.004 (3)
C25A	0.040 (2)	0.105 (4)	0.069 (3)	0.007 (2)	−0.007 (2)	−0.031 (3)
O26A	0.0384 (19)	0.119 (5)	0.120 (3)	0.016 (4)	−0.0075 (17)	−0.068 (5)
C27A	0.063 (3)	0.090 (4)	0.054 (3)	0.020 (2)	−0.004 (2)	−0.030 (3)
C28A	0.0385 (18)	0.088 (4)	0.047 (3)	0.003 (2)	0.004 (2)	−0.009 (3)
C24B	0.047 (2)	0.064 (4)	0.081 (4)	−0.014 (2)	0.002 (3)	0.004 (3)
C25B	0.040 (2)	0.105 (4)	0.069 (3)	0.007 (2)	−0.007 (2)	−0.031 (3)
O26B	0.0384 (19)	0.119 (5)	0.120 (3)	0.016 (4)	−0.0075 (17)	−0.068 (5)
C27B	0.063 (3)	0.090 (4)	0.054 (3)	0.020 (2)	−0.004 (2)	−0.030 (3)
C28B	0.0385 (18)	0.088 (4)	0.047 (3)	0.003 (2)	0.004 (2)	−0.009 (3)

### Geometric parameters (Å, º)

**Table:**

Br1—C22	1.894 (3)	C17—N18	1.321 (3)
S1—O2	1.428 (3)	C17—C22	1.388 (4)
S1—O1	1.428 (3)	N18—C19	1.344 (3)
S1—N8	1.632 (3)	C19—N20	1.343 (4)
S1—C1	1.762 (4)	C19—N23	1.351 (3)
S2—C17	1.759 (3)	N20—C21	1.330 (4)
S2—C10	1.774 (3)	C21—C22	1.359 (4)
C1—C6	1.370 (5)	C21—H21	0.9300
C1—C2	1.382 (5)	N23—C24A	1.448 (6)
C2—C3	1.372 (5)	N23—C28A	1.464 (6)
C2—H2	0.9300	N23—C28B	1.49 (4)
C3—C4	1.373 (5)	N23—C24B	1.58 (4)
C3—H3	0.9300	C24A—C25A	1.508 (8)
C4—C5	1.372 (6)	C24A—H241	0.9700
C4—C7	1.509 (5)	C24A—H242	0.9700
C5—C6	1.380 (6)	C25A—O26A	1.480 (8)
C5—H5	0.9300	C25A—H251	0.9700
C6—H6	0.9300	C25A—H252	0.9700
C7—H71	0.9600	O26A—C27A	1.392 (8)
C7—H72	0.9600	C27A—C28A	1.502 (7)
C7—H73	0.9600	C27A—H271	0.9700
N8—C9	1.421 (4)	C27A—H272	0.9700
N8—H8	0.850 (10)	C28A—H281	0.9700
C9—C10	1.389 (4)	C28A—H282	0.9700
C9—C14	1.392 (4)	C24B—C25B	1.36 (5)
C10—C11	1.385 (4)	C24B—H243	0.9700
C11—C12	1.379 (4)	C24B—H244	0.9700
C11—H11	0.9300	C25B—O26B	0.97 (4)
C12—O15	1.369 (4)	C25B—H253	0.9700
C12—C13	1.381 (4)	C25B—H254	0.9700
C13—C14	1.373 (4)	O26B—C27B	1.65 (5)
C13—H13	0.9300	C27B—C28B	1.47 (4)
C14—H14	0.9300	C27B—H273	0.9700
O15—C16	1.424 (4)	C27B—H274	0.9700
C16—H161	0.9600	C28B—H283	0.9700
C16—H162	0.9600	C28B—H284	0.9700
C16—H163	0.9600		
			
O2—S1—O1	120.06 (17)	C21—N20—C19	115.3 (3)
O2—S1—N8	108.00 (16)	N20—C21—C22	123.5 (3)
O1—S1—N8	105.63 (16)	N20—C21—H21	118.3
O2—S1—C1	106.56 (18)	C22—C21—H21	118.3
O1—S1—C1	108.22 (17)	C21—C22—C17	117.3 (3)
N8—S1—C1	107.88 (14)	C21—C22—Br1	120.4 (2)
C17—S2—C10	100.27 (12)	C17—C22—Br1	122.3 (2)
C6—C1—C2	119.3 (4)	C19—N23—C24A	123.2 (3)
C6—C1—S1	120.9 (3)	C19—N23—C28A	123.1 (3)
C2—C1—S1	119.7 (3)	C24A—N23—C28A	112.9 (4)
C3—C2—C1	120.1 (3)	C19—N23—C28B	113.6 (14)
C3—C2—H2	120.0	C24A—N23—C28B	114.9 (14)
C1—C2—H2	120.0	C19—N23—C24B	115.9 (16)
C2—C3—C4	121.4 (4)	C28A—N23—C24B	112.5 (17)
C2—C3—H3	119.3	C28B—N23—C24B	106 (2)
C4—C3—H3	119.3	N23—C24A—C25A	109.0 (4)
C5—C4—C3	117.6 (4)	N23—C24A—H241	109.9
C5—C4—C7	122.1 (4)	C25A—C24A—H241	109.9
C3—C4—C7	120.2 (4)	N23—C24A—H242	109.9
C4—C5—C6	122.0 (4)	C25A—C24A—H242	109.9
C4—C5—H5	119.0	H241—C24A—H242	108.3
C6—C5—H5	119.0	O26A—C25A—C24A	110.4 (5)
C1—C6—C5	119.4 (4)	O26A—C25A—H251	109.6
C1—C6—H6	120.3	C24A—C25A—H251	109.6
C5—C6—H6	120.3	O26A—C25A—H252	109.6
C4—C7—H71	109.5	C24A—C25A—H252	109.6
C4—C7—H72	109.5	H251—C25A—H252	108.1
H71—C7—H72	109.5	C27A—O26A—C25A	110.7 (6)
C4—C7—H73	109.5	O26A—C27A—C28A	112.2 (5)
H71—C7—H73	109.5	O26A—C27A—H271	109.2
H72—C7—H73	109.5	C28A—C27A—H271	109.2
C9—N8—S1	122.1 (2)	O26A—C27A—H272	109.2
C9—N8—H8	119 (2)	C28A—C27A—H272	109.2
S1—N8—H8	108 (2)	H271—C27A—H272	107.9
C10—C9—C14	117.8 (3)	N23—C28A—C27A	107.8 (4)
C10—C9—N8	121.8 (3)	N23—C28A—H281	110.1
C14—C9—N8	120.4 (3)	C27A—C28A—H281	110.1
C11—C10—C9	121.2 (3)	N23—C28A—H282	110.1
C11—C10—S2	118.0 (2)	C27A—C28A—H282	110.1
C9—C10—S2	120.8 (2)	H281—C28A—H282	108.5
C12—C11—C10	120.0 (3)	C25B—C24B—N23	109 (3)
C12—C11—H11	120.0	C25B—C24B—H243	109.9
C10—C11—H11	120.0	N23—C24B—H243	109.9
O15—C12—C11	124.1 (3)	C25B—C24B—H244	109.9
O15—C12—C13	116.3 (3)	N23—C24B—H244	109.9
C11—C12—C13	119.5 (3)	H243—C24B—H244	108.3
C14—C13—C12	120.4 (3)	O26B—C25B—C24B	119 (5)
C14—C13—H13	119.8	O26B—C25B—H253	107.5
C12—C13—H13	119.8	C24B—C25B—H253	107.5
C13—C14—C9	121.2 (3)	O26B—C25B—H254	107.5
C13—C14—H14	119.4	C24B—C25B—H254	107.5
C9—C14—H14	119.4	H253—C25B—H254	107.0
C12—O15—C16	117.0 (3)	C25B—O26B—C27B	114 (5)
O15—C16—H161	109.5	C28B—C27B—O26B	104 (3)
O15—C16—H162	109.5	C28B—C27B—H273	111.0
H161—C16—H162	109.5	O26B—C27B—H273	111.0
O15—C16—H163	109.5	C28B—C27B—H274	111.0
H161—C16—H163	109.5	O26B—C27B—H274	111.0
H162—C16—H163	109.5	H273—C27B—H274	109.0
N18—C17—C22	121.1 (3)	C27B—C28B—N23	111 (3)
N18—C17—S2	119.5 (2)	C27B—C28B—H283	109.5
C22—C17—S2	119.4 (2)	N23—C28B—H283	109.5
C17—N18—C19	117.3 (2)	C27B—C28B—H284	109.5
N20—C19—N18	125.4 (3)	N23—C28B—H284	109.5
N20—C19—N23	117.8 (3)	H283—C28B—H284	108.1
N18—C19—N23	116.8 (3)		
			
O2—S1—C1—C6	4.6 (3)	C17—N18—C19—N23	179.8 (3)
O1—S1—C1—C6	135.0 (3)	N18—C19—N20—C21	−1.1 (5)
N8—S1—C1—C6	−111.2 (3)	N23—C19—N20—C21	−179.3 (3)
O2—S1—C1—C2	−173.2 (3)	C19—N20—C21—C22	0.1 (5)
O1—S1—C1—C2	−42.8 (3)	N20—C21—C22—C17	0.3 (5)
N8—S1—C1—C2	71.1 (3)	N20—C21—C22—Br1	179.6 (3)
C6—C1—C2—C3	−0.8 (5)	N18—C17—C22—C21	0.1 (4)
S1—C1—C2—C3	177.0 (3)	S2—C17—C22—C21	−179.9 (2)
C1—C2—C3—C4	−1.2 (5)	N18—C17—C22—Br1	−179.1 (2)
C2—C3—C4—C5	2.1 (6)	S2—C17—C22—Br1	0.8 (3)
C2—C3—C4—C7	−177.5 (4)	N20—C19—N23—C24A	−7.8 (5)
C3—C4—C5—C6	−1.1 (6)	N18—C19—N23—C24A	173.8 (3)
C7—C4—C5—C6	178.5 (4)	N20—C19—N23—C28A	−177.4 (3)
C2—C1—C6—C5	1.8 (6)	N18—C19—N23—C28A	4.2 (5)
S1—C1—C6—C5	−176.0 (3)	N20—C19—N23—C28B	−154.3 (14)
C4—C5—C6—C1	−0.8 (6)	N18—C19—N23—C28B	27.2 (15)
O2—S1—N8—C9	−55.5 (3)	N20—C19—N23—C24B	−31.8 (17)
O1—S1—N8—C9	174.9 (2)	N18—C19—N23—C24B	149.8 (16)
C1—S1—N8—C9	59.3 (3)	C19—N23—C24A—C25A	−113.5 (5)
S1—N8—C9—C10	−120.3 (3)	C28A—N23—C24A—C25A	57.0 (6)
S1—N8—C9—C14	61.8 (4)	C28B—N23—C24A—C25A	32.7 (16)
C14—C9—C10—C11	−0.3 (4)	C24B—N23—C24A—C25A	−37 (5)
N8—C9—C10—C11	−178.3 (3)	N23—C24A—C25A—O26A	−55.1 (7)
C14—C9—C10—S2	177.9 (2)	C24A—C25A—O26A—C27A	57.5 (8)
N8—C9—C10—S2	−0.1 (4)	C25A—O26A—C27A—C28A	−59.4 (8)
C17—S2—C10—C11	−70.7 (2)	C19—N23—C28A—C27A	113.4 (4)
C17—S2—C10—C9	111.1 (2)	C24A—N23—C28A—C27A	−57.2 (6)
C9—C10—C11—C12	−2.0 (4)	C28B—N23—C28A—C27A	43 (4)
S2—C10—C11—C12	179.9 (2)	C24B—N23—C28A—C27A	−33.3 (16)
C10—C11—C12—O15	179.7 (3)	O26A—C27A—C28A—N23	58.2 (6)
C10—C11—C12—C13	2.2 (4)	C19—N23—C24B—C25B	−177 (2)
O15—C12—C13—C14	−178.0 (3)	C24A—N23—C24B—C25B	68 (4)
C11—C12—C13—C14	−0.2 (5)	C28A—N23—C24B—C25B	−27 (3)
C12—C13—C14—C9	−2.0 (5)	C28B—N23—C24B—C25B	−50 (4)
C10—C9—C14—C13	2.3 (4)	N23—C24B—C25B—O26B	65 (7)
N8—C9—C14—C13	−179.7 (3)	C24B—C25B—O26B—C27B	−69 (7)
C11—C12—O15—C16	−3.6 (5)	C25B—O26B—C27B—C28B	60 (7)
C13—C12—O15—C16	174.1 (3)	O26B—C27B—C28B—N23	−52 (4)
C10—S2—C17—N18	−3.3 (3)	C19—N23—C28B—C27B	−178.5 (19)
C10—S2—C17—C22	176.7 (2)	C24A—N23—C28B—C27B	32 (3)
C22—C17—N18—C19	−0.9 (4)	C28A—N23—C28B—C27B	−58 (3)
S2—C17—N18—C19	179.1 (2)	C24B—N23—C28B—C27B	53 (3)
C17—N18—C19—N20	1.5 (4)		

### Hydrogen-bond geometry (Å, º)

**Table:**

*D*—H···*A*	*D*—H	H···*A*	*D*···*A*	*D*—H···*A*
N8—H8···O26*A*^i^	0.85 (2)	2.02 (2)	2.846 (8)	163 (2)
N8—H8···O26*B*^i^	0.85 (2)	2.06 (6)	2.895 (5)	165 (3)
C21—H21···N20^ii^	0.93	2.53	3.394 (4)	155
C25*A*—H251···O2^iii^	0.97	2.59	3.377 (6)	138
C27*A*—H272···O2^iii^	0.97	2.52	3.341 (6)	143

Symmetry codes: (i) *x*+1, *y*, *z*; (ii) −*x*+1, −*y*, −*z*+1; (iii) *x*−1/2, −*y*+1/2, *z*−1/2.
